# Radiomic analysis reveals DCE-MRI features for prediction of molecular subtypes of breast cancer

**DOI:** 10.1371/journal.pone.0171683

**Published:** 2017-02-06

**Authors:** Ming Fan, Hui Li, Shijian Wang, Bin Zheng, Juan Zhang, Lihua Li

**Affiliations:** 1 Institute of Biomedical Engineering and Instrumentation, Hangzhou Dianzi University, Hangzhou, China; 2 School of Electrical and Computer Engineering, University of Oklahoma, Norman, Oklahoma, United States of America; 3 Zhejiang Cancer Hospital, Zhejiang Hangzhou, China; Universita degli Studi di Salerno, ITALY

## Abstract

The purpose of this study was to investigate the role of features derived from breast dynamic contrast-enhanced magnetic resonance imaging (DCE-MRI) and to incorporated clinical information to predict the molecular subtypes of breast cancer. In particular, 60 breast cancers with the following four molecular subtypes were analyzed: luminal A, luminal B, human epidermal growth factor receptor-2 (HER2)-over-expressing and basal-like. The breast region was segmented and the suspicious tumor was depicted on sequentially scanned MR images from each case. In total, 90 features were obtained, including 88 imaging features related to morphology and texture as well as dynamic features from tumor and background parenchymal enhancement (BPE) and 2 clinical information-based parameters, namely, age and menopausal status. An evolutionary algorithm was used to select an optimal subset of features for classification. Using these features, we trained a multi-class logistic regression classifier that calculated the area under the receiver operating characteristic curve (AUC). The results of a prediction model using 24 selected features showed high overall classification performance, with an AUC value of 0.869. The predictive model discriminated among the luminal A, luminal B, HER2 and basal-like subtypes, with AUC values of 0.867, 0.786, 0.888 and 0.923, respectively. An additional independent dataset with 36 patients was utilized to validate the results. A similar classification analysis of the validation dataset showed an AUC of 0.872 using 15 image features, 10 of which were identical to those from the first cohort. We identified clinical information and 3D imaging features from DCE-MRI as candidate biomarkers for discriminating among four molecular subtypes of breast cancer.

## Introduction

Breast cancer is the most common disease that affects women globally. Breast cancer subtypes, which are molecular indicators that are indicative of outcome, can be used to guide targeted therapy. A classification system categorizes invasive breast carcinomas into four distinct molecular subtypes, luminal A, luminal B, human epidermal growth factor receptor-2 (HER2)-over-expressing and basal-like, which provide a clinically useful basis for therapeutic selection[1]. The various molecular subtypes have distinct recurrence and survival rates. In particular, the luminal A subtype shows the best survival among the four molecular subtypes [2], while the worst survival is observed for the HER2 and basal-like subtypes [3]. Patients with the luminal B subtype exhibit higher proliferation and have a poorer prognosis than those with the luminal A subtype [4]. Moreover, the basal-like and HER2 subtypes of breast cancer are more sensitive to preoperative chemotherapy [5,6] and are more likely to undergo a pathologic complete response [7] but have a worse prognosis than luminal and normal-like cancers[8,9]. Finally, the basal-like molecular subtype is significantly associated with nodal involvement, which is important for the prognosis of early-stage breast cancer[10].

Molecular subtyping is thus beneficial for the diagnosis and individualized treatment of breast cancer. However, the determination of subtypes by genetic analysis is invasive and expensive, requiring specialized equipment and technical expertise. Immunohistochemical surrogate biomarkers of estrogen receptor (ER), progesterone receptor (PR), and HER2 status have been used to define molecular subtypes. Therefore, demand exists for alternative means of classifying breast cancers into distinct molecular subtypes. Magnetic resonance imaging (MRI) has played an evolving role in the diagnosis and treatment of breast cancer, and studies have highlighted the value of dynamic contrast-enhanced MRI (DCE-MRI) in reflecting the anatomic and functional properties of tumors and facilitating treatment [11–13]. Imaging surrogates are also important for developing personalized treatment and determining tumor heterogeneity when assessing cancer evolution [14,15]. The morphologic and kinetic features extracted from DCE-MRI, including tumor shape, size, spiculation and contrast enhancement, have been shown to be associated with different histological types, tumor grades, and microvessel distributions [11]. In our previous work, we demonstrated that bilateral asymmetry of kinetic features between the left and right breasts [16] and dynamic features in non-tumor breast stroma [17,18] are highly accurate in discriminating benign from malignant tumors.

Over the past few years, pilot studies have attempted to correlate the tumor features extracted from MRI with the molecular subtypes of breast cancer [19,20]; this filed is referred to as “radiogenomics”, while the efforts to discover and use quantitative features are termed “radiomics” [19–21]. Detailed reviews of radiogenomics and radiomics have recently been published elsewhere [22–24]. Many studies have analyzed the associations between qualitative and semi-quantitative MRI features and molecular subtypes [14,19,25] or triple-negative breast cancers [27–29]. Koo et al correlated DCE MRI perfusion parameters (*K*^*trans*^, *k*_*ep*_ and *v*_*e*_) with prognostic factors and the triple negative subtype of breast cancer [30]. Semi-automatically extracted imaging features have also been computed to determine associations between quantitative MRI features and breast cancer subtypes [26,31,32]. The results demonstrated a moderate correlation between two dynamic imaging features and the luminal A and B subtypes of breast cancer [32]. An earlier study of 48 breast cancer patients also identified imaging features associated with the luminal B subtype [31]. That study showed the enhancement dynamics of the tumor along with enhancement of fibroglandular breast tissue, namely background parenchymal enhancement (BPE). A recent study identified 44 MRI features as well as 6 clinical and pathologic features per tumor for classifying molecular subtypes [33]. However, the predictive value of 3D volumetric features from DCE-MRI for the prediction of the four molecular subtypes of breast cancer has not been fully examined.

The purpose of the present study was to investigate the use of features extracted from DCE-MRI and clinical information for the prediction of the molecular subtypes of breast cancer. To our knowledge, our study is the first to classify the four distinct molecular subtypes of breast cancer using 3D volumetric imaging features. In particular, we performed high-throughput extraction of a large number of quantitative and minable imaging features, assuming that the subsets of these features convey prognostic and predictive information. Based on our previous work, we extract imaging features, including the shape, texture, statistics, dynamics and bilateral asymmetry of both the breast lesion and the non-tumor background parenchymal region. To assess the stability of these features for prediction, an additional dataset was used for validation. The successful application of this technique would allow MRI examinations to provide valuable information related to the prognosis of breast cancer.

## Materials and methods

### Patient population

This is a retrospective study approved by the Internal Research Review and Ethical Committee of the Zhejiang Cancer Hospital. All patient information was anonymized before the data were stored in our research dataset. In this study, two cohorts were included to assess and validate the prediction results using the same imaging protocols. For the first cohort, retrospective data from 116 pretreatment breast MRI evaluations performed at Zhejiang Cancer Hospital between January and July 2011 were collected. The first cohort includes 116 pretreatment breast DCE-MRI evaluations acquired from 116 previously underwent breast cancer diagnosis and treatment at this hospital. Forty patients with no pathologic examination or incomplete pathology data and those without a complete MRI sequence were excluded. Patients who underwent breast cancer treatment (e.g., chemotherapy or radiation therapy) before MRI were also excluded (n = 16). Ultimately, 60 patients were analyzed.

A reproducibility cohort of patients was also studied to assess variability. We collected 57 pretreatment breast MRI evaluations, excluding patients who did not receive pathologic examination or had incomplete pathology data (n = 19), as well as those who underwent chemotherapy or radiation therapy before MRI (n = 2). The final dataset included 36 breast cancer patients aged from 29 to 59 years, with a mean age of 47.06 years.

A pathology report was collected from the initial breast biopsy or surgery, and the ER, PR, and HER2 status of each patient with invasive cancer was determined using streptavidin-peroxidase (SP) for immunohistochemistry (IHC) analyses. The ER and/or PR status was determined as positive when at least 1% of the tumor cell nuclei showed staining for the ER or PR [34]. A sample was scored as HER2+ according to the American Society of Clinical Oncology (ASCO)/College of American Pathologists (CAP) guideline recommendations for HER2 testing in breast cancer [35]. The HER2 status was specifically considered positive when the IHC staining intensity score was greater than or equal to three. A HER2 score of 2+, as determined by IHC, with confirmation of gene amplification by fluorescence in situ hybridization (FISH), was also considered to indicate a positive HER2 status. Based on the receptor status, the molecular subtype classification was determined using criteria reported by prior investigators. Four molecular subtypes have specifically been determined: luminal A: ER and/or PR positive, HER2 negative; luminal B: ER and/or PR positive, HER2 positive; HER2: ER and PR negative, HER2 positive; and basal-like: ER, PR, and HER2 negative. Luminal breast cancers were assigned to the luminal B classification if the expression of Ki-67 was at least 14% [1]. The characteristics of the patients in the reproducibility cohort are shown in S1 Table. The statistical test showed no significant differences in clinical characteristics between the subtypes.

### MR image acquisition

The same DCE-MRI scanning method was applied to each woman in our cohort following the current DCE-MRI examination protocol adopted in the Radiology Department of Zhejiang Cancer Hospital. All patients were scanned in the prone position using a Siemens MRI scanner (MAGNETOM Espree-Pink 1.5 T) and a dedicated eight-channel double-breast coil. A precontrast (baseline) series of fat-suppressed T1-weighted and fat-saturated T2-weighted 3D image scans was first acquired, followed by a DCE series after the intravenous injection of a contrast agent at a dose of 0.2 mmol per kilogram body weight and a saline flush of 20 ml at the same flow rate of 2 ml/s. Three postcontrast series (S-0, S-1, S-2 and S-3) of 3D MRI scans and data acquisitions were sequentially performed at time intervals of 1.5 min, 2.5 min and 8.5 min. The parameters were as follows: repetition time (TR)/echo time (TE), 4.4/1.6 ms; flip angle, 12°; acquisition matrix, 512 × 512; slice thickness, 1.2 mm in three MRI scan series (S-0 to S-2) and 0.8 mm in S-3; and spatial resolution, 0.625 × 0.625 × 1.2 mm^3^ in S-0, S-1 and S-2 and 0.625 × 0.625 × 1.2 mm^3^ in S-3. The first three image scan series all had the same number of image slices (88) and the same in-depth resolution, whereas the third series (S-3), with 160 image slices, showed high in-depth resolution. Therefore, only the images acquired from the first three MRI scan series (S-0, S-1, and S-2) were selected and used in the imaging feature computation and analysis.

### Computer-based DCE-MRI analysis

**Fig 1:**
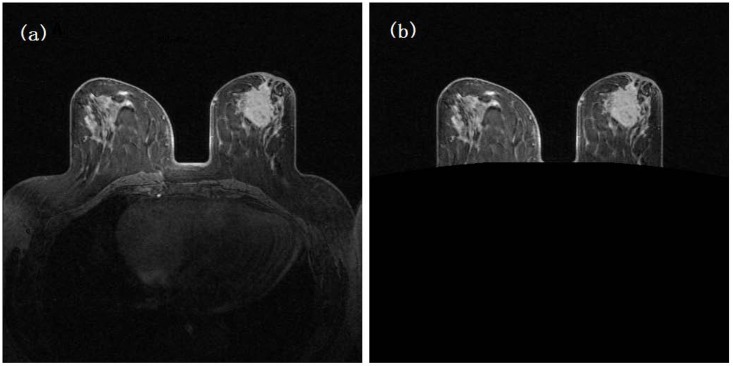
An example of a breast DCE-MRI slice. (a) and its CAD-segmented breast regions depicted on the image slice before contrast agent injection (S-0) in which the non-breast regions behind the chest wall are removed.

Two radiologists with 20 years of experience first annotated the center location of the suspicious breast tumor in each case. The marked tumor center location was initially used as an initial segmentation “seed”, and a volumetric breast tumor boundary contour was automatically segmented [36]. In this method, the segmentation was first conducted with a spatial fuzzy C-means (FCM) algorithm and was then refined using a Markov random field (MRF)-based approach, the parameters of which were adaptively adjusted using segmentation results from contiguous slices. The visual examination and manual correction (if needed) of the segmentation results were performed by our investigators. In this study, manual correction of tumor boundary segmentation was necessary in less than 20% of cases. A computerized scheme was also applied to automatically segment the breast region depicted on all breast MR image slices and to, register sequential MRI scans (S-0, S-1, and S-2) following the same procedure as in our previous study [17]. In brief, the following steps were used for image registration: 1) performing initial image filtering using a Sobel filter, 2) searching for a maximum information window on each image slice, 3) performing an information matching process based on the correlation coefficients of two matched windows, and 4) registering S-1 and S-2 images with the S-0 image through a linearly shifting process. Fig 1 shows an example of CAD-segmented breast regions depicted on the DCE-MRI image slice before contrast agent injection (S-0). The left and right breast areas depicted on each breast MR image slice were segmented to exclude the voxels located behind the chest wall, and a breast tumor was also identified. From the segmented DCE-MRI, we identified the BPE in the abnormal breast exhibiting a breast tumor and the contralateral normal breast in cancer patients.

From the registered MRI sequential scans, we applied a computerized scheme to extract 88 imaging features. The computerized scheme calculated 5 morphologic features, 45 texture features, 15 first-order statistical features, 29 dynamic features from the breast lesion and BPE, and 9 bilateral differences in background parenchymal area. Table 1 summarizes these DCE-MRI features and clinical information.

**Table 1:** Summary of extracted DCE-MR image features.

	Feature number	Description
Clinical information	1–2	Age, menopausal status
Inside tumor	3–43	
First-order statistic	3–17	Skewness, kurtosis, max, average and standard deviation of tumor pixel intensity in precontrast and first and second postcontrast images
Morphologic features	18–22	Volume, diameter, standard deviation of value of tumor radius, roughness, compactness
Texture features	23–37	Contrast, correlation, energy, homogeneity and entropy in precontrast, the first and second postcontrast images
Dynamic features	38–48	Average, standard deviation and maximum value of the subtraction maps (namely, image maps of S-1 –S-0, S-2 –S-0 and S-2 –S-1); mean value of ratio between the subtraction maps (namely, (S-2 –S-0)/ (S-1 –S-0), (S-2 –S-1)/ (S-1 –S-0))
From BPE	49–88	
Texture features	49–63	Contrast, correlation, energy, homogeneity and entropy in precontrast, the first and second postcontrast images
Dynamic features	64–81	Average, standard deviation, and maximum value of the subtraction maps (namely, image maps of S-1 –S-0, S-2 –S-0 and S-2 –S-1)^a^
Bilateral difference in BPE	82–90	Average, standard deviation, and maximum value of three subtraction maps (namely, image maps of S-1 –S-0, S-2 –S-0 and S-2 –S-1)^b^

^a^These features were computed from the abnormal breast exhibiting a breast tumor and the contralateral normal breast in cancer patients.

^b^Bilateral differences between the abnormal breast exhibiting a breast tumor and the contralateral normal breast.

#### Morphologic features

To assess tumor surface irregularity, the intra-tumor variability was measured for each tumor. For this purpose, the radial length was calculated for the distance between a given point on the surface of the tumor and the tumor centroid, and the mean and variance of the radial length were determined. The roughness, compactness, diameter and volume of the breast lesion were also obtained.

#### First-order statistics

To evaluate tumor heterogeneity in terms of the data distribution, histograms were computed using the voxel intensities inside the 3D lesion from which statistical measures of the shape of the histograms were derived. These measures included 1) kurtosis, 2) skewness, 3) variance, 4) minimum, 5) maximum and 6) average values.

#### Texture features

The second-order texture features were assessed using the gray level co-occurrence matrix (GLCM) based on 3D analysis to reflect tumor spatial complexity [37]. Volumetric texture features such as contrast, correlation, energy, homogeneity and entropy were included for both the lesion and the background parenchyma.

#### Dynamic features

Dynamic features were evaluated to assess histopathology and angiogenesis in breast cancer. These features were calculated from the relative average difference in all registered pixel values between image series using the following equation:

$$R_{T}^{S}=\left\{r|r=\frac{I_{T}\left(i\right)-I_{0}\left(i\right)}{I_{0}\left(i\right)},i=1,2,\cdots ,N_{j}\right\},\;T=1,2$$

where *S* represents the regions of interest, including the lesion area and background parenchyma in the normal or abnormal breast; *I*_*T*_(*i*) represents the value of the *i*th-matched pixel voxel in the *T*th image slice; and *N*_*j*_ is the total number of voxels detected inside the 3D volume. The statistical features for the dynamic enhancement of $R_{T}^{S}$ in the breast lesion and background parenchyma were also calculated, including the variances, means, and maximum and minimum values. The background parenchymal features included features that were in the breast lesion and in lesion-free breast areas [16]. The computerized scheme sorted and ranked voxels based on the computed BPE values. Experiments and data analysis were conducted to determine the optimal threshold (p%) to select the percentage of voxels in the top BPE value ranking list to maximize the diagnostic value of using each BPE feature[16]. The contrast enhancement features in the background parenchyma region were computed from the top 1% of voxels in the breast exhibiting a breast lesion and the other, negative (lesion-free) breast.

#### Bilateral difference in BPE

To evaluate breast tissue asymmetry, an important image phenotype that is highly related to the abnormal biological processes leading to cancer development[38], the bilateral asymmetry (p% = 1%) of characteristic kinetic features was also assessed between the abnormal breast exhibiting a breast tumor and the contralateral normal breast. These features were obtained by calculating the differences in the maximum, mean, and variances of ${\text{R}}_{\text{T}}^{\text{s}}$ between the left and right breasts.

### Statistical analysis

All analyses were performed using a publicly available data mining and machine-learning software platform, Weka [39], to conduct feature selection and validation tasks. We employed a multi-class version of the logistic regression implemented in Weka for discrimination of the four molecular subtypes through a one-against-others strategy. Clinical information, namely, age and menopausal status, was also included in the prediction model. The Kruskal—Wallis test was used to test the statistical significance of features selected across the four subtypes. An evolutionary algorithm (EA)-based optimization method, i.e., “EvolutionarySearch”, was used to search for optimal feature subsets for classification. The mutation probability and cross over probability were set at 0.01 and 0.6, respectively. The EA chromosome population in each generation was 500, and the maximum number of generations was 200. To evaluate the performance of the classifier, a receiver-operating characteristic (ROC) analysis was performed, and the area under the ROC curve (AUC) was computed. The EA chromosome that achieved the highest AUC was selected to establish the optimal feature pool and build the optimal classifier.

To avoid overfitting of classifiers to the available data, the leave-one-out cross-validation (LOOCV) test was employed. Specifically, at each iteration of the LOOCV process, one sample was used for testing, and the other sample was used for training. This procedure was repeated for each sample. In each evaluation, we performed a Wrapper Subset Evaluator (WSE) feature selection using all training cases to search for optimal features from the optimal feature subset pool generated from EA. Using these features, a multi-class logistic classifier was trained on the basis of the training set and was tested on the one independent left-out testing case. The importance of the predictive imaging features in the classifier was evaluated by the selection frequencies of features over all of the LOOCV loops. The sensitivity and specificity were calculated using a threshold criterion that was determined as the value that would maximize the average sensitivity and specificity using the best-threshold function in R software, version 3.3.0 (R Development Core Team 2015).

## Results

### Molecular subtype classification in the first cohort

The characteristics of the included patients in the first cohort are shown in Table 2. Sixty women with breast cancer who fulfilled the selection criteria were included in the study sample. All of the patients were of Han Chinese origin, and the median age was 48 years (range: 32–78 years). Among the patients, 35 were premenopausal, and two had a family history of breast cancer. The distribution of the invasive breast cancers by tumor type was as follows: invasive ductal carcinoma (n = 51), intraductal carcinoma (n = 4), mucinous carcinoma (n = 2), carcinoma spongiosum (n = 2) and poorly differentiated adenocarcinoma (n = 1). The subjects included 34 luminal A, 8 luminal B, 7 HER2 and 11 basal-like breast cancer patients. Differences in the categorical variables (menopausal status, family history and tumor type) between distinct molecular subtypes were examined using Fisher’s exact test, and group differences in continuous measures were analyzed using a parametric two-sample independent t-test. The statistical tests showed no significant associations between the molecular subtypes and the pathologic conditions.

**Table 2:** Characteristics of the molecular subtypes of patients.

Characteristic or pathologic condition	All patients (n = 60)	Luminal A (n = 34)	Luminal B (n = 8)	HER2 (n = 7)	Basal-like (n = 11)	P-value
Age	48.8 (32–64)	48.8 (32–62)	48.3 (33–52)	47.6 (43–61)	48.4 (42–64)	0.141
Menopausal status						0.356
Premenopause	35	22	5	2	6	
Postmenopause	25	12	3	5	5	
Family history						
Positive family history	2	2	0	0	0	1.000
No family history	58	32	8	7	11	
Tumor type						0.885
Invasive ductal carcinoma	51	29	7	6	9	
Intraductal carcinoma	4	2	1	0	1	
Mucinous carcinoma	2	1	0	1	0	
Carcinoma spongiosum	2	1	0	0	1	
Poorly differentiated adenocarcinoma	1	1	0	0	0	

**Note:** The P-value for age was obtained from an analysis of variance, and the P-value for menopausal status was obtained using a chi-square test.

To evaluate the effectiveness of DCE-MRI features for the prediction of the molecular subtypes of breast cancer, molecular status was classified using the logistic regression model. The multi-class classifier based on 24 selected features produced an overall AUC value of 0.869. For the luminal A subtype, the classifier using all features computed an AUC value of 0.867 with a 95% confidence interval (CI) of [0.826, 0.908] and an 88.2% classification sensitivity level at 76.9% specificity. Regarding the luminal B subtype, the computed AUC was 0.786 with a 95% CI of [0.585, 0.987], and the optimal sensitivity and specificity were 86.5% and 62.5%, respectively. For the HER2 molecular subtype, the classifier had an AUC value of 0.888±0.083, a sensitivity of 81.1% and a specificity of 100%. Finally, for the basal-like subtype, the computed AUC, sensitivity and specificity were 0.923±0.037, 81.1% and 100%, respectively.

**Fig 2:**
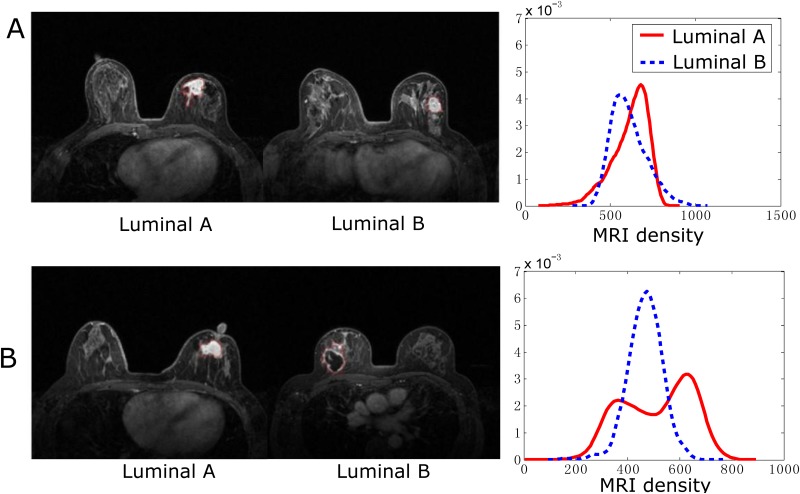
Representative imaging features differentiate tumors with different molecular subtypes. A) Postcontrast MRI of luminal A and luminal B breast cancers. B) Postcontrast MRI of luminal A and luminal B breast cancers and corresponding kurtosis density using a kernel smoothing function.

The Kruskal—Wallis test was performed to evaluate differences in image features among the four subtypes. However, no significant differences among the subtypes were revealed using the Kruskal—Wallis test with a p<0.05. We only observed a trend for an association (p = 0.06) of enhancement between S-2 and S-1 images from the contralateral normal breast. Higher values for this feature were observed in the HER2 subtype than the other subtypes. The importance of each feature representing the frequencies of each feature during the LOOCV loops is shown in Table 3. The top seven features with greater than 50% frequencies in the prediction model included two first-order statistical features (skewness and kurtosis), dynamic features in BPE and in breast lesions, two bilateral asymmetry features and one clinical factor (age). For example, Fig 2 shows that luminal A tumors had lower values of skewness and kurtosis features compared with luminal B tumors.

**Table 3:** List of 24 features that were selected in leave-one-out training and testing cycles to test 60 cases.

Feature	Percentage	Feature	Percentage	Feature	Percentage
F13	100% (60/60)	F89	55% (33/60)	F28	32% (19/60)
F79	98% (59/60)	F39	50% (30/60)	F47	32% (19/60)
F48	95% (57/60)	F74	48% (29/60)	F72	32% (19/60)
F9	88% (53/60)	F87	48% (29/60)	F46	30% (18/60)
F14	88% (53/60)	F68	45% (27/60)	F67	30% (18/60)
F43	78% (47/60)	F72	45% (27/60)	F31	25% (15/60)
F1	70% (42/60)	F29	33% (20/60)	F73	25% (15/60)
F83	68% (41/60)	F42	33% (20/60)	F41	18% (11/60)

**Fig 3:**
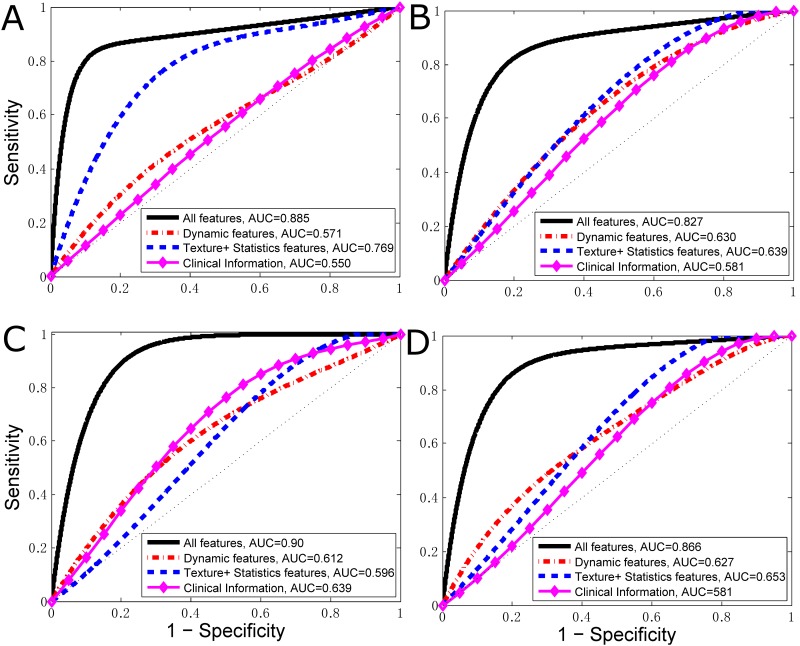
ROC curve in the first cohort for classifying the four molecular subtypes of breast cancer. The classifiers based on dynamic features, morphologic features, first-order statistical features and clinical information are shown. Features are combined to classify between (a) luminal A and non-luminal A tumors; (b) luminal B and non-luminal B tumors; (c) HER2-positive and non-HER2-positive tumors; and (d) basal-like and non-basal-like tumors.

Fig 3 shows and compares ROC curves representing the classification performance levels of applying the 15 dynamic features and the 8 combined texture and first-order statistical features to classify the luminal A, luminal B, HER2 and basal-like tumor molecular subtypes. For the luminal A subtype, the AUC value using the 24 features was significantly increased compared with using dynamic features (p = 0.003) or combined texture and statistical features (p = 0.033). For the luminal B subtype, using the combination of features, the computed AUC was significantly increased to 0.786 compared with using dynamic features (p = 0.04). Regarding the HER2 molecular subtype, the AUC value using all features was 0.888±0.083, which was significantly higher than that using dynamic features (p = 0.001) or combined texture and statistical features (p = 0.045). Finally, for the basal-like subtype, the computed AUC using all features was significantly increased to 0.923 compared with using dynamic features (p = 0.003) or combined texture and statistical features (p = 0.0003). The results indicate that the radiomic features had higher discriminative power in predicting molecular subtypes of breast cancer compared with using only dynamic, texture or statistical features.

### Molecular subtype classification in the reproducibility cohort

Data from the 36 patients in the reproducibility cohort are displayed in S1 Table. The validation dataset included equal numbers (n = 9) of the four subtypes of patients. Among these patients, there were 34 invasive ductal carcinomas, 1 intraductal carcinoma and 1 poorly differentiated adenocarcinoma. The statistical tests showed no significant associations between the molecular subtypes and the pathologic conditions.

The same procedures of feature selection and classification were also performed for the validation cohort. The multi-class classifier based on the 15 selected features yielded an overall AUC value of 0.872. For the luminal A subtype, the classifier using all features computed an AUC value of 0.905 with a 95% CI of [0.7753, 0.9825], a sensitivity of 87.5% and a specificity of 92.9%.. Regarding the luminal B subtype, the computed AUC was 0.835 with a 95% CI of [0.7159, 0.9755], and a 59.3% classification sensitivity level at 100.0% specificity, respectively. For the HER2 molecular subtype, the classifier had an AUC value of 0.947±0.053, and the optimal sensitivity and specificity were 88.9% and 88.9%, respectively. Finally, for the basal like subtype, the computed AUC, sensitivity and specificity were 0.802±0.172, 88.9% and 63.0%, respectively. S1 Fig shows the classification performance by applying 15 image features to classify the molecular subtypes of luminal A, luminal B, HER2 and basal-like tumors, respectively. The Kruskal—Wallis test showed a statistically significant difference in bilateral differences (F83) between subtypes with p = 0.007, as shown in S2 Fig. A higher value for this feature was observed in the basal-like subtype than other subtypes. The importance of each feature representing the frequencies of each feature during the LOOCV loops is shown in Table 4. Among the 15 features, 10 (bold font in Table 4) were identical to those from the first cohort, including 3 texture, 4 BPE, 1 bilateral asymmetry and 2 dynamic features in breast lesions. When the features selected in the first cohort was utilized, multi-class classifier generated an overall AUC value of 0.630 for classifying the four subtypes. After feature selection, the classifier yielded an overall AUC of 0.730 and AUCs of 0.607, 0.807, 0.835, 0.671 for luminal A, luminal B, HER2 and basal-like subtypes, respectively.

**Table 4:** List of 15 image features that were selected in leave-one-out training and testing cycles to test 36 cases in the validation dataset.

Feature	Percentage	Feature	Percentage	Feature	Percentage
**F41**	100% (36/36)	F64	94% (34/36)	**F29**	78% (28/36)
**F47**	100% (36/36)	**F72**	89% (32/36)	**F31**	78% (28/36)
**F83**	100% (36/36)	**F39**	83% (30/36)	**F67**	78% (28/36)
F85	100% (36/36)	**F73**	83% (30/36)	**F28**	50% (18/36)
F8	97% (35/36)	F20	81% (29/36)	F50	50% (18/36)

## Discussion

In this paper, we have presented results showing that features derived from DCE-MRI can be viewed as potential biomarkers for predicting the four molecular subtypes of breast cancer. Radiomic analyses were performed, and the features of morphologic, first-order statistic, texture, bilateral asymmetry and dynamic features in both breast lesions and BPE were extracted and combined for the prediction of molecular subtypes of breast cancer. A multi-class classifier was trained and tested in all the cases, and the AUCs were 0.867, 0.786, 0.888 and 0.923 for the prediction of the luminal A, luminal B, HER2 and basal-like subtypes, respectively, using 24 features. An additional independent validation cohort was also included in our study, and this cohort showed a similar performance with an overall AUC of 0.872. To our knowledge, our study is the first to identify full 3D volumetric DCE-MRI features for the prediction of the four molecular subtypes of breast cancer.

We observed imaging features that predict the distinct molecular subtypes of patients. In our experiments, low kurtosis and skewness were observed for the luminal A subtype, and these features exhibited high importance in the prediction model, which is in line with a previous study that highlighted skewness as a predictor for discriminating molecular subtypes of breast cancer[33]. Kurtosis and skewness features have been identified as biomarkers of tumor heterogeneity [40], and higher values of these biomarkers have been associated with treatment failure [41], while lower values indicate a response to treatment[42]. These findings were consistent with the clinical characteristics of the luminal A subtype, which is less aggressive and has the best prognosis, a high survival rate and a low recurrence rate compared to other subtypes[43–45]. We also observed that the features of dynamic enhancement of the breast lesion and BPE and bilateral asymmetry were among the features of the model. The differences in BPE between the normal and abnormal breasts may reflect the aggressiveness of the breast tumor, which is one of the main features of the HER2 subtype. We also observed that among all the subtypes, HER2 tumors had the highest enhancement values in the normal breasts, while luminal A and luminal B tumors had the lowest values. Compared to the luminal subtypes, the typically more aggressive HER2 subtype is associated with increased neoangiogenesis, which may be responsible for the higher enhancement, even in the normal breast.

Related studies by Mazurowski *et al*. evaluated 23 imaging features, including lesion, texture and dynamic features [31]. They found that the enhancement dynamics of the tumor and background parenchyma were correlated with the luminal B subtype in a 48-patient cohort. A subsequent study from the same group found associations between DCE-MRI features and the luminal A and B subtypes [32]. Similar to previous studies [31,32], our study also identified imaging biomarkers of BPE for prediction. However, these studies did not include the features of skewness, kurtosis and dynamic bilateral asymmetry, which were evaluated as important features for the prediction of subtypes in our study. The predictive nature of skewness and kurtosis is consistent with recently published research for the prediction of three molecular subtypes, namely, luminal-like, HER2-over-expressing and basal-like [33]. The authors examined morphological, histogram-based first-order, and GLCM-based texture features; they obtained an overall accuracy of 71.2% for prediction using imaging features, which was increased to 83.4% when nuclear and histologic grades were incorporated into the prediction model. However, the authors did not examine dynamic or BPE features for prediction, which have been shown as important image biomarkers in prediction. They employed feature evaluation and selection using the ratio of between-group to within-group sums of squares (BW-ratio) test, while our study used a global optimization scheme for radiogenomic feature selection with an AUC value as the fitness criterion. Moreover, the above studies acquired images with 1.5 T and 3.0 T MR systems with different protocols, which may have affected the imaging features and the association results.

In this study, an additional validation cohort was also included to assess variability. The prediction performance of this cohort was similar to that of the first cohort in terms of AUC value, and most of the selected features in the prediction model were the same (10 out of 15). Moreover, the classifier from the validation dataset used similar or identical features as the first dataset, such as skewness, bilateral asymmetric features, and BPE features, which indicates high reproducibility for these image features. However, the features most frequently selected in leave-one-out selection in the first cohort were not among the most selected features in the validation dataset. Therefore, further analyses with more samples should be conducted to validate the radiomic features for classifying subtypes of breast cancer.

This is a preliminary study with several limitations. For example, the statistical power of the study was limited by the relatively small sample size, which was less than in other radiogenomics studies with hundreds of features [46]. Other limitations are that only age and menopausal status were included as clinical information-based parameters in our prediction model; features such as nuclear grade, histologic grade and lymph node status were not used because relevant data were not available for the cohort. Another limitation is that we did not include semi-quantitative or pharmacokinetic analyses of signal intensity time-course data, which is still an important clinical MRI technique of choice for breast cancer diagnosis [47,48]. Despite these limitations, our results are encouraging, as they identify a model using imaging features and menopausal status to predict the luminal A, luminal B, HER2 and basal-like subtypes of breast cancer.

In the present study, imaging features were extracted from DCE-MRI scans of breast cancers. The experimental results demonstrated that the molecular subtype could be predicted based on imaging features from DCE-MRI. Future studies with larger sample sizes examining more radiomic features should be conducted to refine our findings.

## Supporting information

S1 Fig ROC curve in the reproducibility dataset for discriminating the four molecular subtypes of breast cancer. The classifiers based on dynamic features, morphologic features and first-orde r statistic features are shown. Features are combined to classify between (a) luminal A and non-luminal A tumors; (b) luminal B and non-luminal B tumors; (c) HER2-positive and non-HER2-positive tumors; and (d) basal-like and non-basal-like tumors.
(TIFF)

S2 Fig Bilateral asymmetric features between abnormal and normal breast differentiated tumors with four molecular subtypes. (TIFF)

S1 Table Characteristics of the molecular subtypes of patients. (DOCX)
