# Supplementary figures and images for: Radiomic analysis reveals DCE-MRI features for prediction of molecular subtypes of breast cancer
Source: PLoS One. 2017 Feb 6;12(2):e0171683. doi: 10.1371/journal.pone.0171683 (PMC5293281; doi:10.1371/journal.pone.0171683)

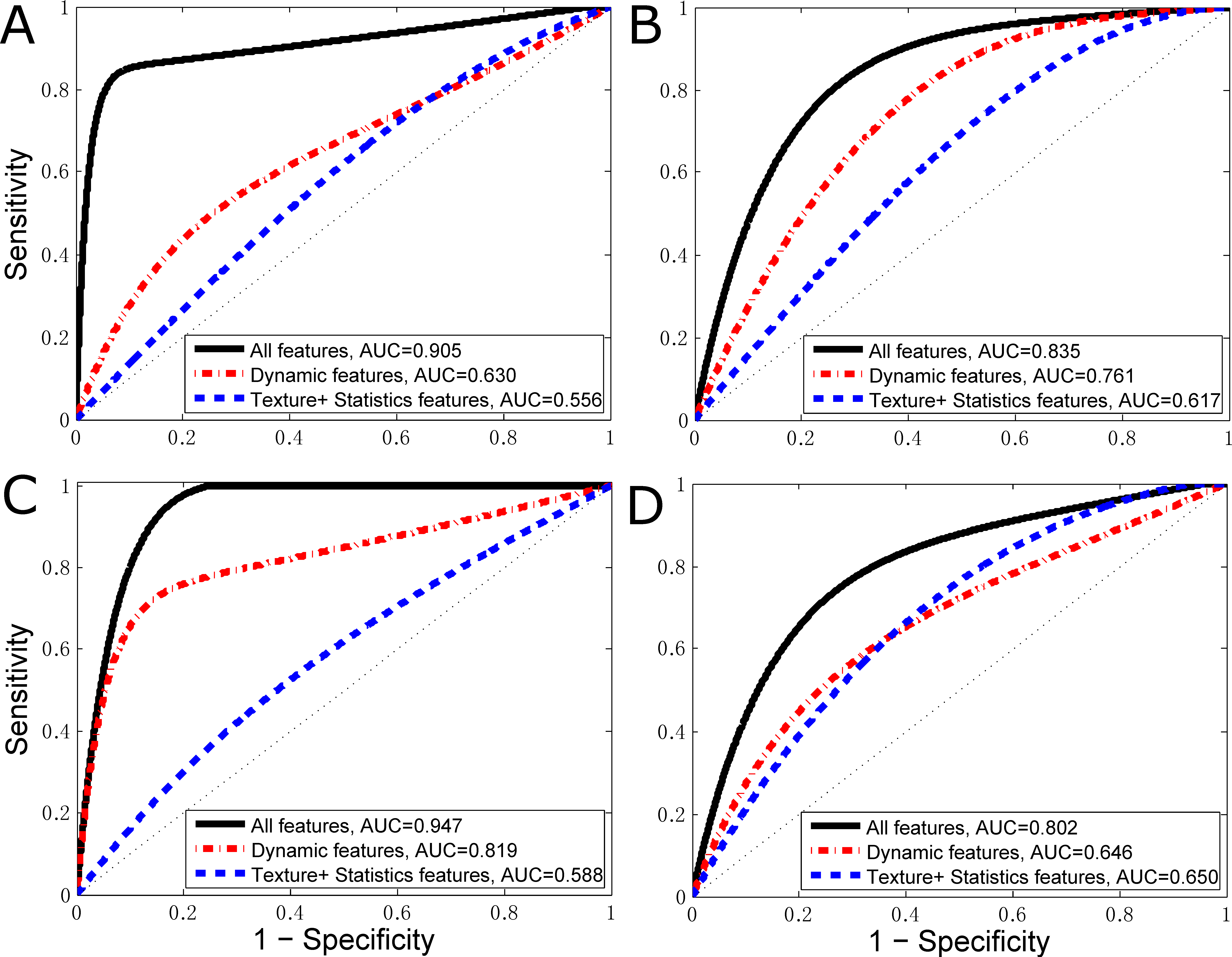

Supplement: S1 Fig — The classifiers based on dynamic features, morphologic features and first-orde r statistic features are shown. Features are combined to classify between (a) luminal A and non-luminal A tumors; (b) luminal B and non-luminal B tumors; (c) HER2-positive and non-HER2-positive tumors; and (d) basal-like and non-basal-like tumors. (TIFF) [file pone.0171683.s001.tiff]

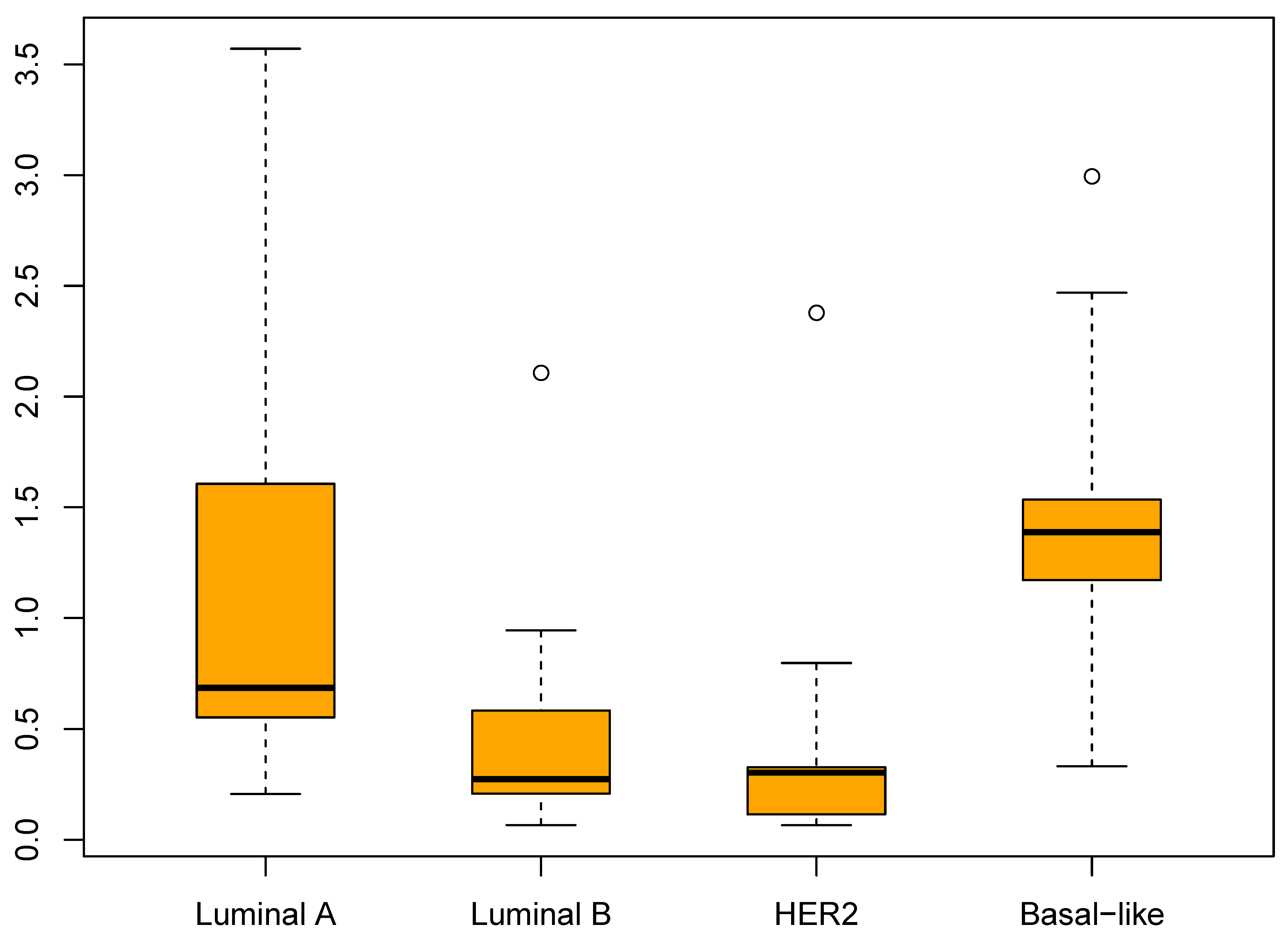

Supplement: S2 Fig — (TIFF) [file pone.0171683.s002.tiff]
