# Supplementary material for: Radiomic analysis reveals DCE-MRI features for prediction of molecular subtypes of breast cancer
Source: PLoS One. 2017 Feb 6;12(2):e0171683. doi: 10.1371/journal.pone.0171683 (PMC5293281; doi:10.1371/journal.pone.0171683)
Supplement: S1 Table — (DOCX) [file pone.0171683.s003.docx]

**S1 Table. Characteristics of the molecular subtypes of patients**

| Characteristic or pathologic condition | All patients (n=36) | Luminal A (n=9) | Luminal B (n=9) | HER2 (n=9) | Basal-like (n=9) | P-value |
| --- | --- | --- | --- | --- | --- | --- |
| Age | 47.06 (29-59) | 48.2 (38-59) | 48.3 (33-52) | 47.6 (43-61) | 48.4 (42-64) | 0.913 |
| Menopausal status |  |  |  |  |  | 0.912 |
| Premenopause | 29 | 7 | 7 | 8 | 7 |  |
| Postmenopause | 7 | 2 | 2 | 1 | 2 |  |
| Family history |  |  |  |  |  | 0.548 |
| Positive family history | 2 | 0 | 1 | 1 | 0 |  |
| No family history | 34 | 9 | 8 | 8 | 9 |  |
| Tumor type |  |  |  |  |  | 0.410 |
| Invasive ductal carcinoma | 34 | 9 | 9 | 8 | 8 |  |
| Intraductal carcinoma | 1 | 0 | 0 | 0 | 1 |  |
| Poorly differentiated adenocarcinoma | 1 | 0 | 0 | 1 | 0 |  |
